# Breast Cancer CAFs: Spectrum of Phenotypes and Promising Targeting Avenues

**DOI:** 10.3390/ijms222111636

**Published:** 2021-10-27

**Authors:** Eiman Elwakeel, Andreas Weigert

**Affiliations:** 1 Institute of Biochemistry I, Faculty of Medicine, Goethe-University Frankfurt, 60590 Frankfurt, Germany; weigert@biochem.uni-frankfurt.de; 2 Frankfurt Cancer Institute, Goethe-University Frankfurt, 60596 Frankfurt, Germany; 3 German Cancer Consortium (DKTK), Partner Site Frankfurt, 60590 Frankfurt, Germany

**Keywords:** cancer-associated fibroblasts, mammary carcinoma, cancer, tumor microenvironment, extracellular matrix, metastasis

## Abstract

Activation of the tumor-associated stroma to support tumor growth is a common feature observed in different cancer entities. This principle is exemplified by cancer-associated fibroblasts (CAFs), which are educated by the tumor to shape its development across all stages. CAFs can alter the extracellular matrix (ECM) and secrete a variety of different molecules. In that manner they have the capability to affect activation, survival, proliferation, and migration of other stromal cells and cancer cell themselves. Alteration of the ECM, desmoplasia, is a common feature of breast cancer, indicating a prominent role for CAFs in shaping tumor development in the mammary gland. In this review, we summarize the multiple roles CAFs play in mammary carcinoma. We discuss experimental and clinical strategies to interfere with CAFs function in breast cancer. Moreover, we highlight the issues arising from CAFs heterogeneity and the need for further research to identify CAFs subpopulation(s) that can be targeted to improve breast cancer therapy.

## 1. Introduction

Breast cancer is the most common cancer in women and a leading cause of cancer related mortality worldwide [1]. Incidence is rising mostly due to improved diagnostics but also due to an increase of life-style-related risk factors such as alcohol consumption, obesity and type 2 diabetes [2]. Mammary carcinoma is categorized into different subtypes depending on estrogen/progesterone receptor expression, human epidermal growth factor receptor 2 (HER2) expression and proliferation measured by Ki67 expression, specifically into luminal A (ER/PR^+^, HER2^−^, Ki67^low^), luminal B (ER/PR^+^, HER2^−^, Ki67^high^; or ER/PR^+^, HER2^+^), HER2 overexpressing (ER/PR^−^, HER2^+^), and triple negative (TNBC) (ER/PR^−^, HER2^−^) breast cancer [3,4]. Breast cancer subtype informs treatment choice, with standard therapy consisting of surgery, (neo-)adjuvant chemotherapy, endocrine or anti-HER2 therapy. With these targeted therapeutic options, local early breast cancer is now considered curable, with the notion that TNBC has the worst prognosis due to limited targeted treatment options [5,6]. Unfortunately, up to 30% of breast cancer patients experience recurrence after primary therapy, mostly in the form of metastasized, advanced breast cancer, which is accompanied by resistance to chemotherapy [7]. Advanced breast cancer is currently regarded as incurable, therefore novel approaches for long-term management are required [8].

High hopes have been invested in strategies targeting the tumor microenvironment (TME), such as immunotherapy. Unfortunately, in patients with metastatic breast cancer, anti-PD-1 therapy has shown little efficacy. This might be owing to the weak immunogenicity driven by the lower mutational load and abundance of tumor-infiltrating lymphocytes [9]. Approaches to combine PD-1 blockade with conventional treatments such as chemotherapy have shown promising results even as first-line treatment in aggressive cancer types such as TNBC by prolonging survival [10]. Nevertheless, response rates were considerably lower compared to other entities [11]. Moreover, therapy resistance in response to immune checkpoint blockade is now established [12]. Thus, new targets in the TME, particularly when considering overcoming therapy resistance, are warranted to improve long-term management of breast cancer. 

Along these lines, fibroblasts are a major component of the TME that has been connected to modulating tumor growth and therapy resistance [13,14,15]. In general, fibroblasts are ubiquitous cells found in every organ. Fibroblasts are, at least at resting stage, spindle-shaped cells that are functionally involved in shaping the stroma, by producing major building blocks of the ECM and modulating their arrangement and density. In addition, their reciprocal communications with neighboring cells enable them to participate in maintaining tissue integrity [16]. Therefore, they determine important structural properties of organs, including elasticity, rigidity and tensile strength. Quiescent or resting fibroblasts found under homeostatic conditions are readily activated once homeostasis is disturbed. For instance, upon wounding they differentiate into wound-associated myofibroblasts under the impact of factors such as transforming growth factor beta (TGF-β), which is the most prevalent fibrogenic growth factor [17,18]. Myofibroblasts are actively proliferating cells that express smooth muscle cell markers (e.g., alpha smooth muscle actin (α-SMA)), enabling them to actively contract wound edges, enhance ECM component synthesis and remodeling to support healing [16]. Pathological hyper-activation of this process, i.e., chronic wound healing, may result in tissue fibrosis [19]. Excessive fibrosis results in distortion of tissue architecture and may finally lead to loss of organ function [20]. Importantly, fibrotic alterations have been connected to cancer emergence, particularly in breast cancer. Mammographical measurements revealed that tumor tissue and tumor-adjacent stroma are 5 to 20 times more rigid than the normal mammary gland [21,22]. Moreover, fibrotic breast disease has been connected to a predisposition towards breast cancer [23]. Given these findings, it is not surprising that activated, myofibroblast-like CAF are found in breast tumors where they continuously contribute to desmoplasia, providing a basis for the phrase that a tumor is ‘a wound that does not heal’ [19,24,25,26]. While it has now been clearly established that CAFs significantly modulate tumor development [27,28,29,30,31], the phenotypic and functional heterogeneity of these cells were less appreciated. Resident fibroblasts themselves are derived from multiple sources, which contribute to their heterogeneity that is further enhanced during tumorigenesis, when a large variety of cells can differentiate into functional fibroblasts [19,32,33]. Therefore, CAFs constitute a diverse cell population with different phenotypes and functions. Targeting one of these phenotypes may be more beneficial for anti-cancer therapy than another, depending on tumor entity, stage and experimental model system [19,32,33,34]. On the following pages, we aim at shedding light on the heterogeneity of CAFs in breast cancer and pointing out current strategies to interfere with their tumor-promoting properties. 

## 2. CAF Sources

Local and distant communications with stromal compartments are essential for cancer cells to nest their developmental niche. One of these stromal cellular compartments is CAFs. Through a reciprocal crosstalk between cancer cells and CAFs, various mechanical and morphological transitions occur affecting tumor progression [35]. The majority of CAFs in breast cancer stroma are derived from resident fibroblasts, which get activated to a myofibroblast-like phenotype. The crosstalk between breast cancer cells and resident fibroblasts promotes induction of a CAF phenotype via Notch signaling [36]. In MCF-7 and Met-1 xenograft models, normal fibroblasts are differentiated to CAFs via endogenous TGF-β and stromal cell-derived factor-1 (SDF-1) in an autocrine manner [37,38]. However, other non-fibroblastic lineages have been described as significant CAF sources. For example, bone marrow-derived mesenchymal stem cells, which are pluripotent stem cells that are involved in tissue remodeling, chronic inflammation, immune response, and cancer progression [39], have been shown to be an important source of CAFs in breast cancer [40]. In addition, fibrocytes, which are circulating bone marrow-derived mesenchymal progenitor cells, have also been suggested as another source of CAFs [41]. Another important source of CAF-like cells is cancer cells undergoing epithelial-to-mesenchymal transition (EMT) giving them a fibroblastic phenotype [42,43]. Adipocytes also contribute to the CAF pool in breast cancer via cancer cell-derived WNT3a [44,45]. Interestingly, standard cancer therapies have been considered to be one functional source of CAFs, as they can activate resident fibroblasts to a CAF phenotype, leading to treatment resistance [46]. In general, the origin of CAFs has been addressed in the past few decades in different types of cancer. However, it remains inconclusive, being dependent on entity and the model system used. Therefore, detailed investigation, including, e.g., lineage-tracing, will be required to resolve the issue of whether any particular subset is decisively involved in breast cancer promotion or suppression.

## 3. CAFs Heterogeneity 

Along breast cancer progression, cancer cells consecutively alter stroma to create a supportive microenvironment. This is in accord with the notion that over 80% of the fibroblasts found in breast tumors display an activated phenotype [47]. Nevertheless, this activation holds a high degree of heterogeneity, which might be owed to their developmental origin and/or functional polarization in the local niche. This always raises the question of when and how fibroblasts become CAFs. The term CAFs implicates that they might acquire this phenotype in established tumors. However, such microenvironmental heterogeneity was found to start already at pre-malignancy and continue throughout cancer progression [48]. In fact, some studies consider it to be a type of progressive developmental education that CAFs acquire across different stages of tumor progression. We recently found that CAFs undergo transcriptomic alteration during tumorigenesis in the transgenic mouse mammary tumor virus–polyoma virus middle T oncoprotein (MMTV-PyMT) model, changing their phenotype from tumor restrictive to tumor promoting [34]. Other studies show that there is a spatial impact on CAFs development, as they get exposed to different tumor-derived mediators, influencing their phenotype. For example, three subtypes of CAFs have been described in the MMTV-PyMT model, which could be spatially segregated and were suggested to be derived from different cellular sources [30]. Another study showed that CAFs situated around the edge of the tumor are phenotypically different from those surrounded by vessels [49]. 

To add a layer of complexity to CAF heterogeneity, a tumor-suppressing CAF phenotype has been described in some studies, which challenges their dogmatic tumor-promoting phenotype. Different studies indicated that cancer cells control the tumor-suppressing capabilities of CAFs. Under certain circumstances, CAFs express TGF-β inhibitors, such as asporin, which is influenced by the tumor genetic subtype. High levels of asporin correlated with good prognosis of luminal ER+/PR+/HER2− tumors, while aggressive tumor subtypes, such as HER2+ and TNBC, expressed low levels of asporin [50]. CD146− CAFs are another subset that suppresses cancer cell proliferation via downregulating ER expression in luminal tumor subtypes [51]. In addition, we identified a transcriptomic signature of a tumor-suppressing CAF phenotype that occurs early during tumor development and correlates positively with breast cancer patient survival [34]. The factors that delineate the tumor-suppressive phenotype of CAFs remain to be identified. Identifying such factors might be helpful to control tumor progression. Across accumulating studies, CAFs heterogeneity is reflected either phenotypically by separation into different subtypes, or functionally depending on their secretome or interaction with neighboring cells. These features are summarized below. 

### 3.1. Phenotypical Heterogeneity (CAF Subtypes)

Phenotypical heterogeneity of CAFs is manifested by different biological markers. Divergent markers that define CAFs in breast cancer are often shared by other stromal compartments or normal counterparts but with a different expression level. For example, markers such as platelet-derived growth factor receptor alpha/beta (PDGFRα/β), CD90/THY1, podoplanin (PDPN), α-SMA, fibroblast-specific protein 1 (FSP-1), fibroblast-activating protein (FAP), fibronectin 1 (FN-1), vimentin (VIM), CD29, CD10 or G protein-coupled receptor 77 (GPR77) are expressed by breast cancer CAFs, although not exclusively. These markers might be segregatedly expressed or co-expressed by different CAF populations. Additionally, differences in CAF marker expression have been observed across different tissues; for example, 10.9% α-SMA+ fibroblasts co-expressed FSP-1 in breast cancer; however, this co-expression was more pronounced (43.5%) in the case of pancreatic cancer [52]. Different subtypes of breast cancer CAFs have been identified in the literature. The earliest CAF subtypes were described by Brechbuhl et al. in human samples, based on CD146 expression. Particularly two subtypes of CAFs in human breast cancer were identified, both with distinct functions. CD146− CAFs suppressed ER expression, while CD146+ CAF promoted tamoxifen sensitivity to the luminal breast cancer cells. They identified a gene signature by bulk RNA-seq, directly influenced by co-culturing fibroblast cell lines with MCF-7 cells (ER+), that reliably predicted recurrence-free survival in patients treated with tamoxifen [51]. Costa et al. identified four CAF subtypes in human breast cancer based on the expression level of α-SMA, and FAP (CAF-S1-S4). Such subtypes were identified by fluorescent-activated cell sorting and were found to be present variously in different subtypes of human breast and high-grade serous ovarian cancers. A significant association between these CAF subsets and breast cancer subtypes was found. For instance, both CAF-S1 and CAF-S4 subsets were preferentially enriched in tumors, while the CAF-S3 subset was significantly accumulated in juxta-tumor areas and CAF-S2 were equally distributed between the two compartments. The CAF-S1 subset was associated with an immune-suppressive environment via secretion of CXCLl12 and enhancing T cell differentiation to regulatory T cells (Tregs), while CAF-S4 subset was lacking such a phenotype [53]. Moreover, two other CAF subtypes were recently identified in human breast cancer patients using single-cell transcriptomic analysis; FSP-1+ CAFs (sCAFs), and PDPN+ CAFs (pCAFs). The ratio between the two subtypes correlates with BRCA mutations in TNBC and clinical outcome [54]. sCAFs were shown to have inflammatory phenotype, while pCAFs had wound healing features. Improved clinical outcome was found to associate with high ratios of sCAF/pCAF. CAFs heterogeneity signifies the notion of a dynamic TME to be capable of keeping track or being shaped by how the tumor evolves. In mice, genetic analyses via microarray and different sequencing approaches helped revealing more complexity and heterogeneity of CAFs. Cremasco et al. identified two CAF subtypes in 4T1 and 4T07 subcutaneous models of breast cancer by microarray; FAP+PDPN+ CAFs and FAP+PDPN− cancer-associated pericytes (CAPs). Both subtypes were spatially and functionally different. For instance, PDPN+ CAFs were abundant in dense ECM and were associated with immunosuppression, whereas PDPN- CAPs were mainly localized around vasculature and had no immunosuppressive function [49], somewhat contradicting the human CAF data obtained by Friedman et al. [54]. Another three subtypes of CAFs were identified by single cell transcriptomic analysis of MMTV-PyMT-derived CAFs and validated in patient data sets via bioinformatical comparison by Bartoschek et al. The three subtypes were vascular CAFs (vCAFs) that particularly expressed Nidogen2, matrix-related CAFs (mCAFs) that were Pdgfrα+, and developmental CAFs (dCAFs) that were Pdgfrβ− and Scrapie Responsive Gene 1 (Scrg1)+. α-SMA and FAP did not segregate these subtypes, as shown by Costa et al. [53]. mCAF and vCAF were associated with metastasis; however, only mCAF correlated negatively with a prognostic signature [30]. Along the same lines, we identified two CAF subtypes in MMTV-PyMT mice by bulk RNA sequencing: early-stage CAFs and late-stage CAFs. Late-stage CAFs had an inflammatory phenotype characterized by activated nuclear factor kappa B (NF-κB) signaling and an enrichment of vCAF markers, whereas early-stage CAFs showed higher levels of nuclear exclusion of NF-κB. Interestingly, the transcriptomic signature of the early-stage CAFs correlated with early stage in human breast tumors and improved survival of breast cancer patients [34]. All these studies indicated that divergent CAF subtypes identified across different animal models or human breast cancer samples, sometimes come with an assumingly different outcome. However, more complex description of subtypes using more and new molecular markers might dissolve these discrepancies and help to identify the link between these subtypes. Identification of CAF subtype-specific markers may open avenues to target such subtypes individually by tailored compounds, but needs to be connected to functional analysis. 

### 3.2. Functional Heterogeneity

As mentioned earlier, CAF heterogeneity might correlate with their function in different types of malignancy. To understand this heterogeneity, the key features distinguishing resident fibroblasts from CAFs must be first considered. As such, ECM remodeling, immune and vascular regulation, metabolic adaptation reshape the stroma as a function of the CAF secretome or mutual crosstalk with different cells. In this regard, CAFs are a significant source not only of ECM components, but also of cytokines, chemokines, extracellular vesicles, and metabolites that influence tumor development. 

#### 3.2.1. ECM Remodeling

ECM is the non-cellular stromal component of which the architecture impacts many cellular functions. It is composed of glycosaminoglycans and structural as well as matricellular proteins that coordinate cell communication, adhesion and movement [19]. Solid tumors, particularly those derived from glandular epithelium such as breast cancer, are associated with desmoplasia, which is a pronounced stiffened stroma that comprises a substantial proportion of the tumor mass, and fosters aggressive behavior of cancer cells [55]. Perpetual remodeling of the ECM is a hallmark of CAFs, usually rendering the ECM more stiff by excessive deposition of type I and III collagens (Col-I, III) and degradation of type IV collagen (Col IV) [13,19]. Excessive deposition of ECM and secretion of matrix metalloproteinase (MMP) enzymes create a self-sustaining feed-forward loop of CAF activation and ECM remodeling to generate a more stiffened ECM, resulting in biomechanical and biochemical changes that affect tumor growth and invasion [56,57]. For example, Col-I, secreted mainly by CAFs, enables disseminated breast cancer cells to migrate towards blood vessels to intravasate into the blood stream [58]. Additionally, constitutive collagen cross-linking is another feature of ECM remodeling stimulated by CAFs via the cross-linking enzyme lysyl oxidase (LOX) [59,60]. LOX is expressed during early stages of breast carcinogenesis by CAFs, whereas in later stages it is also induced in hypoxic cancer cells, promoting invasiveness [61]. The resulting stiff ECM promotes cell invasion via integrin clustering, focal adhesion kinase (FAK) phosphorylation and Rho GTPase activation [62]. Human breast cancer cells can migrate faster on stiffer stroma, and their persistent migration can be directed following a stiffness gradient, through a process termed “durotaxis”. Human breast cancer displays a stiffness gradient as shown by atomic force microscopy. As such, the tumor is generally stiffer than surrounding tissue with the invasive front being the stiffest [63]. This is coherent with CAFs mostly localizing peripherally, surrounding tumor nests [64,65]. Moreover, mechanical stress induced by a stiff ECM activates YAP/TAZ signaling in cancer cells perturbing their actin cytoskeleton and promoting their aggressiveness [66]. Histopathological analysis of MMTV-PyMT tumors shows nuclear accumulation of YAP in invasive carcinoma compared to hyperplasia and normal mammary tissues [67]. Furthermore, stiffened ECM contributes significantly to increase interstitial fluid pressure, which constitutes a barrier for therapeutic delivery of drugs inside the tumor [68].

#### 3.2.2. Immune Regulation

The tumor-associated immune system is a critical regulator of tumor growth [69], and not only shaped by the tumor cells themselves, but also by CAFs. Immune regulation by CAFs is either direct via secretion of different molecules (cytokines or chemokines), or indirect via ECM remodeling that controls intratumoral infiltration. Divergent CAF phenotypes have a significant share of regulating the immune landscape in cancer, sustaining an immune-suppressive tumor-promoting status [70,71,72]. Among these phenotypes are FAP+ PDPN+ CAFs, which are immunosuppressive and regulate cytotoxic T cell localization and motility via a nitric oxide (NO)-dependent mechanism [49]. Costa et al. also found that FAP_high_ CAFs are correlated with Treg-mediated immunosuppression and a poor outcome of breast cancer patients [53]. Apart from FAP+ cells, PDGFRα+ CAFs were found to support differentiation of tumor-associated macrophages (TAMs) towards an immunosuppressive M2 phenotype via the secreted glycoprotein chitinase-3-like-1, which is involved in fibrosis and chronic inflammation [73]. In another study, FSP-1+ CAFs recruited TAMs to tumors via CXCL14 induced nitric oxide synthase 1 (NOS1) [74]. Interestingly, CAFs were found to indirectly influence the immune response via deposition of ECM and remodeling the matrix on which immune cells localize or migrate. For instance, TAM infiltration was improved by the extensive deposition of Col-I and hyaluronic acid by α-SMA+ CAFs [63]. Better defining the role of specific CAF subtypes in orchestrating the immune interactions may be crucial for improving current immunotherapies [75].

#### 3.2.3. Vascular Regulation

Angiogenesis and lymphangiogenesis, the formation of new blood and lymph vessels respectively, are essential for cancer cells to prevail. Angiogenesis is critical for tumor progression to survive the limited nutrients, while both angiogenesis and lymphangiogenesis are invasive processes to help cells colonize distant sites. In early stages of breast cancer MDA-MB-231 and MDA-MB-435 xenograft models, CAFs have been shown to orchestrate neovascularization in strict dependence on NF-κB activation [76]. Late stage MMTV-PyMT tumors however displayed a massive accumulation of CAFs with vascular and pro-metastatic functions compared to early stage, which was independent of NF-κB signaling [34]. CAF-derived CXCL12 recruited endothelial progenitor cells in a co-implantation xenograft model with MCF-7 cells and fibroblasts [77]. Hypoxic CAFs were found to promote endothelial sprouting in breast cancer through pronounced vascular endothelial growth factor (VEGF) signaling [78]. CAFs enhanced lymphangiogenesis in the MMTV-PyMT model by promoting hyaluronic acid-expression [79]. In 4T1 xenograft model, CAF depletion decreased expression of pro-angiogenic factors, such as VEGF, PDGFR and granulocyte-macrophage colony-stimulating factor (GM-CSF), and resulted in suppression of angiogenesis and lymphangiogenesis [80]. These studies suggest the potent role CAFs play in promoting breast cancer angiogenesis and lymphangiogenesis.

#### 3.2.4. Metabolic Adaptations

Cells in the TME are often under metabolic stress due to hypoxia and nutrient deprivation. Cancer cells produce energy to overcome nutrient deprivation via different survival pathways, including the “Reverse Warburg” pathway and autophagy. The Warburg effect is the adaptation of tumor cells to a low-oxygen situation, involving conversion of glucose into lactate rather than pyruvate [81,82]. Later, a different model was proposed describing metabolic coupling of cancer cells with CAFs to support their massive and uncontrolled proliferation and nutrients demand, which is called the “Reverse Warburg”. In this model, cancer cells induce oxidative stress in CAFs, which in turn undergo a metabolic switch to glycolysis providing energy-rich lactate and pyruvate to metabolically support adjacent cancer cells [83]. Loss of caveolin-1 (CAV-1) is a characteristic feature of breast cancer CAFs, resulting in increased aerobic glycolysis and gain of a myofibroblastic phenotype [84,85,86]. Supporting this notion, a metabolic switch towards glycolysis in breast cancer CAFs was observed upon downregulation of a subunit of the isocitrate dehydrogenase 3 complex (IDH3a), which helped maintain a CAF phenotype [87]. Along these lines, the direct contact of breast cancer cells with CAFs transferred G protein-coupled estrogen receptor 1 (GPER) to the cytoplasm. Cytoplasmic GPER induced aerobic glycolysis in CAFs via cyclic adenosine monophosphate (cAMP)-dependent protein kinase A/cAMP-response element binding protein (PKA/CREB) signaling [88]. Conversely, depletion of FAK in breast cancer CAFs was associated with enhanced glycolysis in cancer cells via activation of PKA [89]. Autophagy is another survival pathway, which is induced by hypoxia-inducible factor 1-α (HIF1-α) to sustain cellular functions via degradation of cytoplasmic constituents, recycling of ATP, and the maintenance of cellular biosynthesis [90,91]. HIF-1 stabilization in CAFs leads to mitophagy and, in turn, shifting towards aerobic glycolysis [84]. The autophagic properties of breast cancer CAFs are involved in enhancing stemness and metastatic potential of breast cancer via Wnt/β-catenin or via Toll-like receptor 4 [92,93,94]. CAFs metabolic coupling with cancer cells remains to be heterogeneous, and unraveling its key parameters could pave a new avenue for breast cancer treatment. 

#### 3.2.5. Tumor Stemness and Chemoresistance

Cancer stem cells (CSCs), are a particularly tumorigenic and chemoresistant population in tumors [95]. CAFs have been implicated in promoting chemoresistance and cancer stemness in multiple tumor types [96,97,98], including breast cancer [99,100]. For example, breast cancer CAFs can modulate tamoxifen resistance in breast cancers via activation of the phosphatidylinositol 3-kinase/protein kinase B (PI3K/AKT) and mitogen-activated protein kinase/extracellular signal-regulated kinase (MAPK/ERK) pathways and phosphorylation of ERα at serine 118 [101,102]. α-SMA+VIM+ CAFs also induced breast cancer stemness via periostin-dependent Wnt signaling [100]. Another very interesting study by Su et al. found that CD10+ GPR77+ CAFs subpopulation is responsible for breast cancer stemness and chemoresistance via sustained secretion of NF-κB-dependent IL-6 and IL-8 [103]. IL-7-expressing CAFs were identified as another subpopulation that sustains breast cancer stemness via CXCL12 secretion [104]. Co-culturing CAFs with breast cancer cells induced stemness via CCL2-mediated Notch signaling pathway [105]. Excessive release of high-mobility group box 1 (HMGB1) by autophagic CAFs was found to enhance stemness of luminal breast cancer cells and resistance to doxorubicin [106,107]. In multiple types of cancer, including breast cancer, cytotoxic stressors, such as chemotherapy or radiotherapy, enrich cancer cells with CSC features that contribute treatment resistance and tumor relapse [108,109]. Such stressors also perpetuate metabolic and phenotypic transformation of resident fibroblasts into a CAF phenotype that promotes CSC features [46]. For example, CD44+CD24− CSCs were enriched in breast cancer after neoadjuvant chemotherapy. Additionally, chemotherapy-altered α-SMA+ CD90+ CAFs secrete pro-stemness chemokines, such as CXCL1, CXCL2, CXCL5, and CXCL6 [110]. Moreover, taxmoxifen activates G-protein-coupled receptors on breast cancer CAFs, promoting their proliferation via the GPER/EGFR/ERK axis [111,112]. Another study showed that MCF7 breast cancer cells co-cultured with fibroblasts show induced resistance to tamoxifen and fulvestrant [113]. 

Together, these studies indicate that CAFs might be an integral part of cancer stemness and therapy resistance promotion, which should be considered for therapy decision making.

#### 3.2.6. Motility and Invasiveness

One key feature of breast cancer malignancy is metastasis towards secondary organs. Accumulating clinical and experimental data support the hypothesis that CAFs regulate cell motility and metastastatic spread via EMT [114]. EMT is an epigenetic programming of cancer cells that gives them a more motile mesenchymal phenotype, increasing their invasive potential [19,115]. This enables cancer cells to intravasate blood vessels and circulate within the blood stream. Circulating cancer cells are effectively resistant to anoikis [116]. Survival within the bloodstream is ensured through traveling covered with fibrin-fibronectin clots (emboli) and/or platelets [117]. Once circulating cancer cell extravasate, their colonizing potential and survival is dependent on the ECM and the microenvironment of the distal site [118,119]. CAFs induce EMT in breast cancer cells via TGF-β1 signaling [120]. CAF-derived CXCL12 was also found to enhance migration and invasion capacity of breast cancer cells [121]. Senescent mammary fibroblasts were found to increase motility of co-cultured mammary epithelial cells via Rac exchange factor, Tiam1, and the integrin-binding phosphoglycoprotein osteopontin [122]. Additionally, collective metastasis was induced when CAFs remodeled the ECM into tracks enriched with Col-I, promoting protrusion of cancer cells [123]. In the same framework, CAFs were found to dictate metastasis direction of breast cancer cells. For instance, TNBC normally tends to metastasize viscerally. However, TNBC cells have shown pronounced skeletal metastasis when grown with CAF-derived CXCL12 and insulin-like growth factor (IGF) [124,125]. Apart from the secretory features of CAFs in inducing EMT, CAFs were found to induce heterophilic physical interactions with cancer cells inducing cooperative invasion. For instance, heterotypic physical interactions between cancer cells and CAFs induce β-catenin recruitment, α-catenin/vinculin interaction, and actin remodeling. This allows CAFs to exert an intercellular physical force on cancer cells and promote cooperative tumor invasion [126]. Interestingly, not only CAFs contribute to EMT induction in breast cancer. It has been found that normal fibroblasts at the interface areas have a greater capacity in modulating breast cancer cells compared to CAFs [127]. 

#### 3.2.7. Genetic and Epigenetic Alterations

The genomic landscape of CAFs is debated in the literature. Several studies have described CAFs as genetically stable cells compared to cancer cells, making them an attractive target for therapeutics [128,129,130]. For example, breast cancer CAFs displayed copy number variation (CNV) and p53 mutation in only one tumor out of twenty-five samples [131]. In another study, comprehensive molecular characterization of breast cancer CAFs showed no genetic alterations, despite the difference in gene expression between CAFs and normal fibroblasts [32,132]. On the contrary, other studies have shown genetic alterations in CAFs including CNVs and loss of heterozygosity (LOH). For example, 17 CNVs were detected in CAFs of MDA-MB-231 and MDA-MB-435s xenograft models, including amplifications and deletions by oligonucleotide microarray analysis [133]. LOH was identified in the mammary stroma of micro-dissected tissues of 11 human breast samples, several of which were exclusively stromal incidents [134]. LOH or complete loss of p53 is another particular example of CAFs genomic modification, especially that LOH of p53 in CAFs contributes to resistance to radiotherapy and chemotherapy [135]. LOH of p53 was also identified in micro-dissected fibroblastic stromal cells of breast cancer, and associated with regional lymph-node metastases in sporadic breast cancer [136]. Concerning the issue of genetic alterations in CAFs, the EMT potential of cancer cells, developing towards a CAF-like phenotype should be considered [42,43]. Addition of these cells to the CAF pool will certainly result in the observation of CNVs, LOH and mutations within the CAF population. 

Epigenetic modifications activating resident fibroblasts to a myofibroblastic phenotype are reversible during acute inflammation. However, such modifications are putatively irreversible in cancer stroma [19]. That explains why CAFs show a persistent phenotype in vitro even without cancerous stimulation [77]. Multiple types of epigenetic modifications were observed in breast cancer CAFs, including DNA methylation and histone acetylation. Such modifications lead to a dynamic shift in CAF phenotype sustaining a feed-forward loop of CAF activation [37]. Albrengues et al. demonstrated that aberrant histone acetyltransferases and DNA methyltransferases induced by the proinflammatory cytokine leukemia inhibitory factor (LIF) sustained CAF phenotypes of multiple cancer types including breast cancer [137]. DNA methylation in breast cancer stroma correlated significantly with HER2 expression in 143 human breast tumors, suggesting that it might attribute to specific biological features of HER-2-positive tumors [138]. Distinct methylation profiles were also observed in epithelial and myoepithelial cells and stromal fibroblasts from normal breast tissue and breast carcinomas, implying the role they play in changing the TME along tumor development to promote invasion [139].

Regulation of the epigenetic machinery observed in CAFs is, among others, controlled by micro RNAs (miRNAs). miRNAs are small non-coding RNAs that can be reciprocally delivered between cells in the TME, and can directly silence the expression of tumor suppressor genes and induce genomic instability via modulating enzymes that affects DNA methylation and histone modifications [140,141,142]. For example, *miR-221/222* directly suppressed ER expression, which was significantly associated with reduced recurrence-free and overall survival of breast cancer [143]. A different study showed that reduced expression of CAF-derived *miR-26b* from ER+ breast tumors was associated with enhanced cancer cell migration [144]. Along the same lines, *miRNA-200* family members were also downregulated in breast cancer CAFs. *miRNA-200* family members and their targets impacted expression of α-SMA, FN-1, contributed to ECM remodeling and inhibited tumor initiation and invasion [145]. In addition, downregulation of *miRNA-205* contributed to acquisition of a CAF phenotype via YAP1 expression [146]. Moreover, downregulation of *miRNA-320* was essential for fibroblasts to acquire a tumor promoting phenotype via loss of phosphatase and tensin homolog (PTEN) [147]. On the other hand, *miRNA-9* upregulation was shown to activate resident fibroblasts into CAFs [148]. These studies provide a strong body of evidence that epigenetic regulation is a major principle affecting the interaction of CAFs and cancer cells. However, further studies are required to elucidate if the genetic integrity of CAF-like cells that are not derived from cancer cells is altered. 

## 4. CAFs and Prognosis

The gene signature of the tumor stroma can be regarded as a prognostic tool in many types of cancer. One of the first studies that addressed the prognostic relevance of stroma in breast cancer patients was conducted by Finak et al. In this study, the authors presented a stromal gene signature that predicted poor outcome in multiple subtypes of breast cancer [149]. Along the same line, features of desmoplastic stroma can also offer a prognostic tool. For example, aggressive HER2+ and TNBC lesions have stiffer stroma associated with high expression of linearized collagens, compared to less aggressive luminal A and B subtypes [63]. Additionally, linearized and stiffened collagen bundles were found to be predictive of poor breast cancer patient prognosis [150]. However, these studies were not specific to CAFs, since these features may have been influenced by other cells in the tumor stroma as well [151]. Given that CAF density correlates positively with most desmoplastic cancer types, other studies identified specific CAF-derived prognostic signatures. One of these studies demonstrated that higher levels of procollagen-lysine, 2-oxoglutarate 5-dioxygenase family members, which are LOX required for production of structural components of the ECM, were detected in breast cancer, compared to normal mammary tissue [152]. Other studies have shown that downregulation of the tumor suppressive fibroblast-derived SLIT or their roundabout homologue 1 receptor activates proliferative WNT signaling and was associated with poor prognosis [153,154]. On the other hand, downregulation of PDGFRα was associated with poor prognosis of breast cancer patients [40]. In fact, a PDGFRα_low_ PDGFRβ_high_ CAF subset was identified as a marker for ductal carcinoma in situ (DCIS) [36].

The prognostic relevance of CAFs includes chemoresistance prediction, which can aid in clinical decision-making regarding proposed treatment protocols. This owes, on the one hand, to the positive correlation of CAFs with desmoplasia. For example, high density of α-SMA+ CAFs is correlated with resistance to neoadjuvant chemotherapy in breast cancer [103]. Additionally, presence of CD146+ CAFs predicts tamoxifen sensitivity in ER+ breast cancer patients [51]. In addition, a fibroblast-related gene signature of 50 differentially expressed genes predicts resistance to neoadjuvant chemotherapy in breast cancer [155]. Moreover, detection of circulating CAFs in liquid biopsy samples confers a dynamic prognostic tool during cancer progression. This tool demonstrates that circulating FAP and α-SMA expressing CAFs were present in 88% of breast cancer patients with metastases, 23% of patients with localized disease and 0% of healthy donors [156,157]. On the other hand, as mentioned earlier, chemotherapy modulates CAF signaling to sustain cancer stemness and in turn chemoresistance. Signaling pathways activated in CAFs upon chemotherapy could be targeted to serve as a supplemental diagnostic tool to select patients for anti-CAF/CSC therapies. For instance, levels of phosphorylated signal transducer and activator of transcription 1 (pSTAT-1) in CAFs, which induces pro-stemness chemokines following chemotherapy, may help clinicians to decide to implement CAF-directed therapies [39,110].

## 5. Targeting of CAFs for Cancer Therapy

Rapid development of drug resistance, genetic diversity, and spatial distribution of cancer cells might be problematic for developing targeted therapeutics in desmoplastic tumors. By contrast, CAFs can provide an attractive target for breast cancer therapeutics, both at the primary and secondary site. As discussed above, CAFs are genetically more stable and less likely to acquire drug resistance compared to cancer cells. Moreover, spatial distribution of CAFs within the desmoplastic stroma is another advantage. For instance, CAFs are mostly localized peripherally to cancer cell nests surrounding them, or in proximity to blood vessels, making them more accessible for therapeutic systemic diffusion [30,68]. 

**Figure 1:**
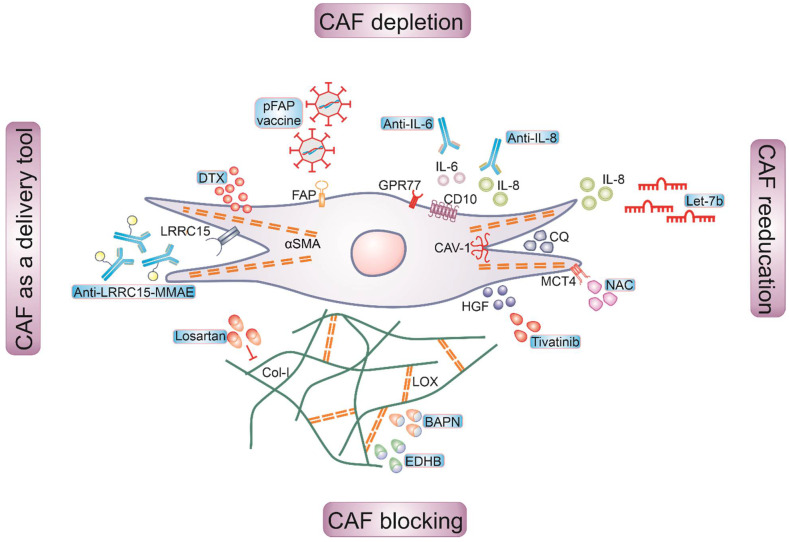
Schematic representation of therapeutic targeting strategies of breast cancer CAFs. Four general approaches that target CAFs for cancer therapy include CAF depletion, re-education, blocking and using CAFs as a delivery tool. Depleting α-SMA via docetaxel conjugated nanoparticles (DTX), eliminating FAP via DNA vaccine, or neutralizing antibodies targeting IL-6 and IL-8 were used to deplete CAFs. CAF re-education is approached to acquire a dedifferentiated phenotype through downregulating MCT-4 via N-acetyl-cysteine (NAC) administration, restoring the expression of Cav-1 via chloroquine (CQ) treatment, or overexpressing tumor suppressor *Let-7b* miRNA. CAF functions were blocked by inhibiting extracellular matrix (ECM) proteins, such as Col-I and LOX, or CAF-derived signals such as HGF. Expression of Collagen Type I (Col-I) can be inhibited via Losartan treatment, while LOX expression can be inhibited via, ethyl 3,4-dihydroxybenzoate (EDHB) or β-aminopropionitrile (BAPN). HGF expression can be inhibited via Tivantinib treatment. CAFs were used to target a cytotoxic payload toward tumor, such as conjugating antimitotic drug, monomethyl auristatin E (MMAE), to anti-LRRC15 antibody to target its delivery to CAF-rich tumors.

Over the past decade, different studies targeted the tumor-promoting functions of CAFs directly via CAF depletion or reprogramming towards a normal fibroblast phenotype, or indirectly via targeting CAF interactions with other neighboring cells [158] (Figure 1). Some of these studies have been translated into clinical trials. However, sole CAF-targeting therapeutics did not achieve much success in clinical trials, probably due to the heterogeneity of CAFs outlined above and the lack of specific markers. CAFs rather emerged as an important complement to multiple immune therapies. 

### 5.1. Depleting CAFs

Given the profound impacts of CAFs on the tumor progression, depleting CAFs in the tumor stroma provided a viable option towards attenuating their impact. For example, depleting α-SMA+ CAFs, using docetaxel-conjugated nanoparticles, reduced lung metastases in 4T1 and MDA-MB-231 models [159]. Along the same lines, novel immunotherapies have been used to deplete FAP+ CAFs in different breast cancer models. A DNA vaccine targeting FAP attenuated expression of proangiogenic factors such as VEGF, PDGFR and GM-CSF and suppressed angiogenesis and lymphangiogenesis in a 4T1 breast cancer model [80]. CD10+ GPR77+ CAFs were also depleted in a patient-derived xenograft model via neutralizing antibodies targeting IL-6 and IL-8 that were abundantly expressed by these cells. CD10+ GPR77+ CAFs depletion efficiently delayed tumor onset and restored chemosensitivity to docetaxel [103].

### 5.2. Reeducating CAFs

Targeting CAF activation to revert them into a deactivated status emerged as another interesting strategy of CAF targeting. For instance, CAFs express lower levels of the tumor suppressor miRNA *Let-7b* compared to their normal fibroblast counterparts. This was also connected to CAF differentiation, since re-expression of *Let-7b* in human breast cancer CAFs reduced their cancer promoting capabilities [97,158,160]. CAF reprogramming was also tested clinically and showed efficacy. For example, Cav-1 expression was restored in breast cancer CAFs upon treatment with chloroquine, which is an antioxidant and autophagy inhibitor [84]. Based on this study, a clinical trial was launched termed Preventing Invasive Breast Neoplasia. At this trial, a reduction in proliferation DCIS lesions and enhanced immune cell migration into mammary ducts was observed upon chloroquine administration prior to surgical excision [161]. N-acetyl-cysteine (NAC) is another antioxidant that has been used clinically to reprogram breast cancer stroma via downregulating the expression of the gycolytic marker monocarboxylate transporter 4 (MCT4). A pilot clinical study by Monti et al. demonstrated that NAC administration decreased cancer cell proliferation rates in women with stages 0 and I breast cancer [162]. Whether these effects were strictly due to targeting CAFs remain unclear.

### 5.3. Blocking CAF Functions

Another therapeutic strategy to target CAFs is via inhibiting CAF-derived signals that influence cancer development. For example, CAFs were shown to secrete high levels of hepatocyte growth factor (HGF), which activates its cognate receptor, c-Met kinase, on cancer cells, promoting tumor development [156]. Targeting HGF by the c-Met inhibitor Tivantinib demonstrated early signs of anti-tumor activity when combined with Gemcitabine in a phase 1 trial of multiple solid tumor patients including breast cancer patients [160]. This trial was warranted for phase 2 and 3, but only in locally advanced or metastatic non-small cell lung cancer [163,164]. Col-I in CAFs was also inhibited by the anti-fibrotic agent Losartan, leading to delayed tumor progression of multiple cancer models, including MMTV-PyMT breast cancer model [165]. LOX also emerged as a potential target for inhibiting CAF-derived signals. The LOX inhibitor ethyl 3,4-dihydroxybenzoate (EDHB) was found to decrease tumor fibrosis and metastasis in a MDA-MB-231 xenograft model [166]. The LOX inhibitor β-aminopropionitrile (BAPN) was used to block LOX expression in the MMTV-PyMT model, leading to decreased ECM stiffening and delaying tumor progression [60]. Nevertheless, these attempts to block CAF functions might not be specific, because of the redundancy in the cells expressing the targeted molecules [167,168]. 

### 5.4. CAFs as a Drug Delivery Tool

Using CAFs as a means for drug delivery comprises an appealing strategy to evade CAFs heterogeneity. This strategy was applied by Purcell et al. who identified Leucine-rich-repeat-containing 15 (LRRC15) as a new surface marker for CAFs in multiple solid tumors including breast, head and neck, lung, pancreatic cancer. They conjugated the antimitotic drug, monomethyl auristatin E, to anti-LRRC15 humanized IgG1 antibody to target its delivery to CAFs via a protease cleavable valine–citrulline. Reduced tumor growth was observed upon using this therapeutic strategy in xenograft models of breast cancer, NSCLC-adenocarcinoma, osteocarcinoma and glioblastoma. However, breast cancer xenografts displayed tumor regrowth after treatment [169], indicating potential development of treatment resistance. 

## 6. Conclusions 

It has become increasingly clear that CAFs play an important role in cancer cell integrity and tumor dynamics not only in the mammary gland, but also in other tumor entities [13,15,156,158]. As discussed above, the prognostic and therapeutic significance of CAFs in cancer therapy has become apparent. Importantly, targeting CAFs may help to overcome major issues remaining in long-term breast cancer management, namely distant metastasis and treatment resistance. However, a major caveat is the heterogeneity these cells display during tumor development over time, in different breast cancer subtypes and in individual tumors simultaneously in different microenvironmental niches. The goal must hereby be to selectively target CAFs with tumor-promoting characteristics, while leaving those with anti-tumor properties unaffected. Characterizing CAF heterogeneity is therefore a critical first step, which has been approached by recent studies using systems biology approaches in single cells. This could be delineated first through CAFs lineage tracing which is crucial to deconvolute the factors driving phenotypical and functional heterogeneity. The next critical steps will be manipulating the drivers of CAF heterogeneity to be able to avoid (trans)-differentiation of other cells to CAFs once tumor niches have been emptied of pro-tumor CAF subsets, and identifying specific and hopefully non-redundant functions associated with distinct phenotypes. This could be done using models that closely mimic human breast cancer such as patient-derived xenografts, e.g., in humanized mice, or in tumor organoids interacting with patient-derived fibroblasts. Such models should avoid the pitfalls of 2D culture, and consider the impact of ECM and CAFs spatial location on tumor development [170]. This could be achieved by different approaches, including engineering ECM protein-based scaffolds using 3D bioprinting to mimic the spatial conditions of the TME [171,172]. Such printed scaffolds may constitute a gradient hydrogel that allow investigating the influence of ECM stiffness in directing cell invasion [173]. Using microfluidic platforms on 3D models, adds another layer of precise control to the experimental setup, and provides a better understanding of tumor-stroma interactions [174]. These approaches may then inform decisions which CAFs subset(s) to target in which way, yielding another arrow in the quiver of anti-tumor therapy.

## Data Availability

No new data were created or analyzed in this study. Data sharing is not applicable to this article.
